# Agrivax: A chemical inducer of plant immunity identified by high-throughput screening

**DOI:** 10.1016/j.abiote.2026.100038

**Published:** 2026-03-19

**Authors:** Changlong Liu, Qingqiu Shi, Ning Bai, Xuechun Hu, Yajin Ye, Lijuan Zhou

**Affiliations:** National Key Laboratory for the Development and Utilization of Forest Food Resources, Co-Innovation Center for Sustainable Forestry in Southern China, Nanjing Forestry University, Nanjing, 210037, China

**Keywords:** Small molecule, Chemical screening, Plant immunity, Agrivax

## Abstract

Plants possess a multilayered immune system that detects and responds to pathogen invasion through pattern-triggered immunity (PTI) and effector-triggered immunity (ETI). We recently developed a high-throughput screening platform using transgenic *Arabidopsis thaliana* expressing the *β-glucuronidase* (*GUS*) reporter gene under the control of the promoter of the PTI marker gene *FRK1* (*pFRK1::GUS*) to identify chemical inducers of plant immunity. Among the ∼10,000 screened compounds, we identified 10 candidate compounds with the potential to activate *FRK1* expression. In this study, we focused on one of these small molecules, designated Agrivax, for analysis. Agrivax is a previously unreported small molecule that activates immune signaling. Agrivax induces *FRK1* expression in a dose-dependent manner, triggers the transient phosphorylation of MAPKs, and enhances resistance against *Pseudomonas syringae* pv. tomato DC3000 (*Pst* DC3000). While Agrivax alone did not elicit reactive oxygen species (ROS) production, it synergistically amplified flg22-and chitin-induced ROS bursts and root growth inhibition. Furthermore, Agrivax pretreatment potentiated the ETI-associated hypersensitive response upon challenge with *Pst* DC3000 carrying *AvrRpt2*. These findings demonstrate that Agrivax functions as an immune priming chemical to activate immune responses and disease resistance, making it a promising lead compound for developing plant immune inducers and a valuable tool for dissecting immune signaling pathways.

*Dear Editor,*

During the long-term co-evolutionary “arm races” between plants and their pathogens, plants have developed a sophisticated, multilayered defense system against diverse biotic challenges, including microbial pathogens, herbivores, and parasitic plants [1]. Plant cells employ cell-surface pattern recognition receptors (PRRs) to detect conserved molecular patterns, such as pathogen-associated molecular patterns (PAMPs), thereby activating pattern-triggered immunity (PTI) [2]. In response, pathogens secrete effector proteins to manipulate the host immune system and thus promote infection [3]. To counteract those pathogen effectors that suppress plant immunity, plants have evolved intracellular nucleotide-binding leucine-rich repeat receptors that recognize specific effectors and initiate a stronger immune response known as effector-triggered immunity (ETI) [[3], [4], [5], [6]]. The activation of PTI and ETI typically induces a cascade of downstream defense responses, including a reactive oxygen species (ROS) burst, the influx of calcium ions, activated mitogen-activated protein kinase (MAPK) signaling, and transcriptome reprogramming [7,8].

Plant immune elicitors are biological or chemical molecules, including proteins, oligosaccharides, lipids, small metabolites, plant hormones, and their derivatives, that mimic pathogen invasion or tissue damage signals to activate innate immunity. Protein-based elicitors are currently the most extensively studied and best-characterized elicitors [9]. For instance, flg22, a 22-amino acid peptide derived from bacterial flagellin, is widely used as a model elicitor in plant immunity research [10]. flg22 originates from the conserved N-terminal region of flagellin and is specifically recognized by FLAGELLIN SENSING 2 (FLS2) at the plant cell membrane. FLS2 recruits the co-receptor BRI1-ASSOCIATED KINASE 1 (BAK1) to form an active receptor complex, initiating downstream immune signaling [11]. This recognition triggers typical PTI responses [12,13]. Similarly, chitin, a fungal cell-wall component, activates defense responses via the receptors CHITIN ELICITOR RECEPTOR KINASE 1 (CERK1), LYSM-CONTAINING RECEPTOR-LIKE KINASE 4 (LYK4), and LYK5 [14]. Oligosaccharide elicitors, comprising short carbohydrate chains of 2–10 monosaccharides linked by glycosidic bonds, have emerged as potent inducers of disease resistance in crops. Upon recognition by cell-surface PRRs, representative oligosaccharides such as β-1,3-glucans activate early immune events [15]. Decursin, a recently discovered immune inducer, is a natural furanocoumarin compound extracted from the roots of plants from the *Angelica* genus. The plasma-membrane proteins AtCERK1, AtLYK4, and AtLYK5 perceive decursin in Arabidopsis (*Arabidopsis thaliana*), triggering a transient ROS burst, MAPK activation, and the expression of immune response genes such as those encoding PAMPs [16].

Advances in the study of immune elicitors are driving innovations in sustainable plant protection strategies. “Plant vaccines” based on elicitors can induce broad-spectrum resistance and diminish reliance on synthetic pesticides. To identify inducers of plant immunity, we recently developed a reporter-based high-throughput chemical screening system. *FRK1* (*flg22-INDUCED RECEPTOR-LIKE KINASE 1*), a highly conserved marker gene with early expression in the PTI signaling pathway, is widely present across various plant species [17]. We placed the *β-glucuronidase* (*GUS*) gene under control of the *FRK1* promoter and used it to generate transgenic *Arabidopsis* *pFRK1::GUS* seedlings [16]. Using this transgenic line, we performed high-throughput screening against compound libraries comprising approximately 10,000 small molecules. After three rounds of validation, we identified 10 candidate compounds with the potential to activate *FRK1* expression [16].

In this study, we selected one of these small molecules for characterization. This chemical, designated as Agrivax, has a structure distinct from that of known immune inducers (Fig. 1A, Fig. S1). *FRK1* expression in transgenic *Arabidopsis* *pFRK1::GUS* leaf discs was induced by Agrivax in a dose-dependent manner (Fig. 1H). Reverse-transcription quantitative PCR (RT-qPCR) of transgenic *pFRK1::GUS* *Arabidopsis* seedlings treated with Agrivax for 8 h confirmed that Agrivax induces *FRK1* expression (Fig. 1D). The combination of Agrivax and flg22 led to substantially higher *FRK1* expression compared to individual treatments (Fig. 1D).

**Fig. 1:**
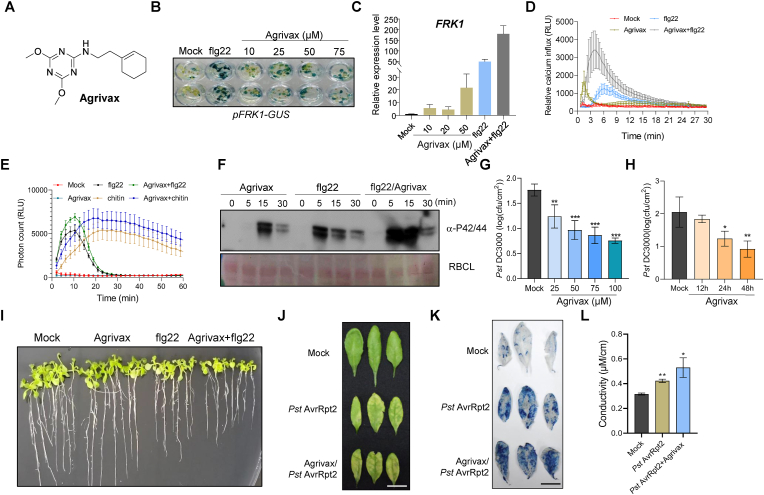
Identification and characterization of the plant immune inducer Agrivax. **A** Chemical structure of Agrivax. **B** Agrivax induces transient cytosolic calcium influx and synergistically enhances flg22-triggered calcium transients. Calcium levels were monitored using Aequorin-based luminescence (RLU) in 7-*d*-old *Arabidopsis* seedlings expressing the aequorin gene (Col-A seedlings). Values represent means ± standard error of the mean (SEM; *n* = 8 biological replicates). **C** Quantification of Agrivax-induced reactive oxygen species (ROS) production in *Arabidopsis*. Col-0 seedlings were treated with 50 μM Agrivax for 60 min; 1% (v/v) DMSO was used as a mock control. Values represent means ± SEM (*n* = 8 biological replicates). **D** RT-qPCR analysis of *FRK1* expression. Col-0 seedlings were soaked in 10, 20, or 50 μM Agrivax for 8 h before analysis, Meanwhile, in the Agrivax and flg22 co-treatment group, 50 μmol of Agrivax was used for treatment, and 100 nmol of flg22 was added 2.5 hours before sampling. Values represent means ± SEM (*n* = 3 biological replicates). **E** Cell death kinetics quantified by measuring electrolyte leakage (conductivity). Leaf discs were incubated in deionized water, and conductivity was measured using a digital conductometer; ion leakage confirms the potentiation of the hypersensitive cell death response. Values represent means ± SEM from three independent biological replicates (*n* = 3). Asterisks indicate significant differences as determined by -tailed Student's *t*-test (∗, *P* < 0.05; ∗∗, *P* < 0.01). **F** Dose-dependent repression of *Pst* DC3000 growth in leaves pre-treated with Agrivax for 24 h. Bacterial titers were quantified 2 days post-inoculation (dpi). Asterisks indicate significant differences compared to Mock (∗∗, *P* < 0.01; ∗∗∗, *P* < 0.001, 2-tailed Student's *t*-test; *n* = 5 biological replicates; values represent means ± SEM). **G** Time-course of Agrivax-induced resistance, estimated based on *Pst* DC3000 growth. Leaves were treated with Agrivax at 12, 24, or 48 h prior to inoculation with *Pst* DC3000. (∗, *P* < 0.05; ∗∗, *P* < 0.01, 2-tailed Student's *t*-test; *n* = 5 biological replicates; values represent means ± SEM). **H** Agrivax activates *FRK1* expression. Transgenic *pFRK1::GUS* seedlings grown for 5 days on half-strength MS medium were soaked in the indicated concentrations of Agrivax for 8 h, followed by GUS staining to detect *FRK1* expression; 100 nM flg22 served as a positive control, and DMSO was used as the solvent control. **I** Agrivax activates MAPKs. *Arabidopsis* seedlings were soaked in 20 μM Agrivax, followed by immunoblotting with an anti-p44/p42 antibody; 100 nM flg22 served as a positive control. Ponceau S staining was conducted as the loading control. **J** Agrivax enhances cell death triggered by *Pst AvrRpt2*. Leaves from 4-week-old *Arabidopsis* plants were injected with 50 μM Agrivax, followed by inoculation with *Pst AvrRpt2* 24 h later. Leaf status was observed 24 h post inoculation; DMSO was used as a control. Scale bar, 1.5 cm. **K** Trypan blue staining to visualize dead cells. *Arabidopsis* leaves were treated as in J. Scale bar, 1.5 cm. **L** Agrivax enhances flg22-imposed inhibition of root growth. Seedlings were treated with 50 μM Agrivax and/or 100 nM flg22, and root length was examined following a 5-day growth period in half-strength MS medium; 1% (v/v) DMSO was used for the control. Seven biological replicates were performed. Scale bar, 2 cm.

The finding that Agrivax activated plant immune–responsive gene expression suggested that Agrivax triggered upstream immune events, ultimately leading to the induction of immune-related gene expression in the nucleus. To test this hypothesis, we examined the effects of Agrivax on the induction of other hallmarks of plant immunity, namely calcium influx, ROS bursts, and MAPK activation, specifically MAPK3 and MAPK6. To precisely quantify calcium dynamics, we employed an aequorin-based bioluminescence reporter system [16]. Agrivax treatment of 5-day-old *Arabidopsis* seedlings expressing the aequorin gene (Col-A) elicited a distinct, transient calcium spike (Fig. 1B). Notably, when the seedlings were treated with Agrivax together with flg22, calcium influx further rose, far exceeding the response to either stimulus alone (Fig. 1B). While Agrivax alone did not induce ROS accumulation, Agrivax treatment together with flg22 or chitin substantially amplified the ROS burst elicited by flg22 or chitin alone (Fig. 1C), suggesting that Agrivax potentiates PAMP-mediated immune responses.

We analyzed the activation of MAPK over 30 min following Agrivax treatment. Agrivax triggered the rapid, transient phosphorylation of MAPKs; the signals began to fade at 30 min (Fig. 1I). This MAPK activation pattern resembles that observed following treatment with PAMPs but not effectors, which elicit prolonged, sustained MAPK activation. When plants were treated with Agrivax together with flg22, MAPK phosphorylation was markedly enhanced (Fig. 1I). MAPK activation results in transcriptome reprogramming of the plant immune signaling pathway, a phenomenon that aligns with the effects of Agrivax, which activates both responses. To assess the effects of Agrivax on phytopathogen resistance, we inoculated the leaves of *Arabidopsis* plants with the bacterial pathogen *Pseudomonas syringae* pv. tomato DC3000 (*Pst* DC3000). Pre-treatment with Agrivax conferred robust protection against later *Pst* DC3000 infection, as evidenced by significantly lower bacterial proliferation compared to the mock-treated controls (Fig. 1F–G). This Agrivax-induced resistance followed clear dose- and time-dependent kinetics, suggesting highly regulated priming of the plant innate immune system (Fig. 1F–G).

Enhanced immunity caused by PAMPs (such as flg22) can significantly inhibit root growth in *Arabidopsis* [18,19]. Indeed, Agrivax treatment modulated this growth–defense tradeoff in a dose-dependent manner. While 50 μM Agrivax had no discernible effects on seedling vigor, higher doses (75 and 100 μM) significantly restricted root growth (Fig. S2). Notably, when co-administered with flg22, Agrivax intensified the growth inhibition imposed by the peptide (Fig. 1L), a phenotype that aligns with the amplified ROS burst and MAPK activation under this treatment. These results further support the role of Agrivax in synergistically enhancing plant immune responses in conjunction with flg22.

Accumulating evidence indicates that PTI potentiates ETI during bacterial infection [2]. The transient, rapid MAPK activation in response to Agrivax treatment indicates that it is an immune-priming chemical. To determine whether Agrivax also enhances ETI, we treated *Arabidopsis* plants with 50 μM Agrivax for 24 h, followed by inoculation with *Pst* DC3000 (*AvrRpt2*). Compared to control plants inoculated with the pathogen alone, plants pretreated with Agrivax exhibited a significantly stronger hypersensitive response, indicating that Agrivax enhances ETI-associated immune responses (Fig. 1J). We validated this enhancement by trypan blue staining (Fig. 1K) and electrolyte leakage assays (Fig. 1E), confirming that Agrivax significantly potentiates the programmed cell death typically elicited by AvrRpt2 recognition. Taken together, these findings identify Agrivax as a new synthetic small molecule that functions as a potent immune elicitor, bridging and amplifying multiple layers of the plant immune system.

Our results contribute to our understanding of elicitor-triggered plant immunity. Like the known PAMPs flg22 and chitin, Agrivax activated early PTI markers, including *FRK1* expression and MAPK phosphorylation. However, unlike typical PAMPs, Agrivax alone did not induce a ROS burst, suggesting it may selectively activate specific branches of the PTI signaling cascade. Moreover, our observation that Agrivax enhances ETI supports the recent hypothesis that PTI provides a baseline level of disease resistance that facilitates ETI amplification, as proposed by Ngou et al. [2]. (2022).

Despite these promising results, several important questions require further investigation. First, the molecular target(s) of Agrivax are unknown, limiting our understanding of its action. Notably, Agrivax-induced MAPK activation remained unaltered in mutants of key PRRs, including *fls2*, *cerk1*, and *lyk4 lyk5* (Fig. S3), suggesting that Agrivax may function through a different PRR. Second, the efficacy and specificity of Agrivax across different plant species and against diverse pathogens have not been explored. Third, potential trade-offs between enhanced immunity and plant growth or yield under field conditions were not assessed. Finally, the chemical stability, environmental persistence, and possible toxicity of Agrivax require evaluation. From a translational perspective, the physicochemical properties of Agrivax present specific challenges; its solubility is lower in water than in organic solvents such as ethanol and DMSO (Table S1). Future research should prioritize structure–activity relationship (SAR) studies to engineer Agrivax analogs with enhanced bioactivity and optimized solubility. Addressing these parameters, along with chemical stability, environmental persistence, and toxicological profiles, will be essential for its use in sustainable agriculture. Nonetheless, Agrivax represents a potent chemogenetic probe for dissecting the complexities of plant innate immunity. Beyond its utility as a research tool, it holds significant promise as a lead compound for developing next-generation, high-efficiency plant immunity inducers.

## 1 Materials and methods

### 1.1 Chemical libraries and screening

Chemical libraries were purchased from TargetMol and Selleck. Dimethyl sulfoxide (DMSO, CAS 67-68-5) was purchased from Shanghai Yuanye Biotechnology. To identify modulators of plant innate immunity, a recently developed high-throughput chemical screening platform was employed using an *Arabidopsis thaliana* reporter line. This line carries the *β-glucuronidase* (*GUS*) reporter gene under the control of the *FRK1* promoter (*pFRK1::GUS*). In the primary screen, transgenic seedlings were treated with a diverse chemical library at a working concentration of 50 μM. To ensure the robustness of the hits, a three-tier validation pipeline was employed. The first round of validation confirmed direct induction of *FRK1* expression at 50 μM to eliminate false positives. The second and third rounds of screening focused on establishing dose-response profiles. Only lead candidates exhibiting potent agonistic activity at concentrations as low as 10 μM were selected for functional characterization.

### 1.2 Plant materials and growth conditions

Wild-type *Arabidopsis thaliana* (accession Columbia-0, Col-0) and the *pFRK1::GUS* transgenic line were used as plant materials. Unless otherwise specified, all plants were cultivated in an environmentally controlled growth chamber at 20–25 °C with a 16-h light (40 μmol m^−2^ s^−1^)/8-h dark photoperiod.

*Arabidopsis* seeds were surface-sterilized in a solution of 0.02% (v/v) Tween-20 and 30% (v/v) sodium hypochlorite with agitation at room temperature for 15 min before being rinsed five times with sterile deionized water. The surface-sterilized seeds were stored at 4 °C for 2–3 days to break dormancy and promote synchronized germination. The stratified seeds were sown in Petri plates containing half-strength Murashige and Skoog (MS) basal medium (pH 5.7) supplemented with 0.5% (w/v) sucrose and solidified with 0.4% (w/v) Phytagel and grown vertically in the growth chamber. After 7-12 days, the seedlings were used directly for experimental treatments or transplanted to soil for further cultivation.

### 1.3 GUS staining

GUS staining was performed as previously described using the *pFRK1::GUS* transgenic line [16]. Five-to seven-day-old *pFRK1::GUS* seedlings were treated with Agrivax (or DMSO as a control) at 37 °C for 6–8 h, vacuum-infiltrated with GUS staining solution containing 100 mM NaH_2_PO_4_ (pH 7.0), 10 mM Na_2_EDTA, 0.5 mM K_4_[Fe(CN)_6_]·3H_2_O, 0.5 mM K_3_[Fe(CN)_6_], 0.1% (v/v) Triton X-100, and 1 mM X-Gluc (5-bromo-4-chloro-3-indolyl-β-d-glucuronic acid cyclohexylammonium salt), and incubated in the dark at 37 °C for 12–16 h. After staining, chlorophyll was removed by repeated destaining with 95% (v/v) ethanol until the tissues became transparent. The samples were then photographed.

### 1.4 RNA extraction and RT-qPCR

Total RNA was extracted from plant tissues using an UltraPure RNA Kit (Cowin Biotech, China) according to the manufacturer's instructions. The integrity of the RNA was verified by agarose gel electrophoresis, and its concentration was measured using a NANA-ONE Ultra Micro spectrophotometer (YOONING, China). One microgram of total RNA was treated with genomic DNA removal reagent and reverse-transcribed into cDNA using a HisScript II Q RT SuperMix Kit (Vazyme, China, Item Number: R333-C1). RT-qPCR was performed on a Bio-Rad CFX Connect™ real-time PCR detection system with ChamQ Universal SYBR qPCR Master Mix (Takara). The relative expression levels of the target genes were calculated using the 2^−ΔΔCt^ method, with *EF1α* (AT1G07940) serving as the internal reference gene. All experiments included three biological replicates and three technical replicates.

### 1.5 MAPK activation assay

*Arabidopsis* seedlings grown on half-strength MS medium for 7 days were treated with Agrivax solution or DMSO (mock control) for the indicated durations. After treatment, the samples were gently blotted dry with paper towels and immediately frozen in liquid nitrogen. The frozen tissue was ground into a powder, lysed in an appropriate volume of protein extraction buffer (50 mM Tris-HCl, pH 7.5, 200 mM NaCl, 1% [v/v] Triton X-100), and incubated on ice for 5 min. The lysates were centrifuged at 12,000×*g* for 10 min at 4 °C. The supernatant was mixed with an appropriate volume of 5 × protein loading buffer and heated at 80 °C for 10 min in a water bath. The samples were resolved by 4–20% (w/v) SDS-PAGE and transferred onto a PVDF membrane. The membrane was blocked with 5% (w/v) skim milk in TBST for 1 hour. and immunoblotted with rabbit monoclonal anti-phospho-p44/42 MAPK antibody (Cell Signaling Technology; diluted 1:2000) to detect phosphorylated MAPKs.

### 1.6 Bacterial infection assay

For pathogen growth assays, fully expanded leaves of 4-week-old *Arabidopsis* plants were infiltrated with *Pseudomonas syringae* pv. *tomato* (*Pst*) DC3000 suspended in 10 mM MgCl_2_ to an OD_600_ of 0.002 using a needleless syringe. Infected plants were maintained under high humidity conditions. Three leaf discs (0.5 cm in diameter) were collected from each leaf 2 days after infection, surface-sterilized in 75% (v/v) ethanol for 30 s, rinsed twice with sterile distilled water, and homogenized in 10 mM MgCl_2_. Serial dilutions of the homogenate were plated on King's B (KB) agar medium containing 50 μg/mL rifampicin. Following incubation at 28 °C for 48 h, colony-forming units (CFUs) were counted. In each experiment, a minimum of nine independent plants were analyzed per treatment, and three independent biological replicates were performed.

### 1.7 Measuring ROS production

Seven-day-old Col-0 seedlings were placed in a 96-well plate containing 100 μL double-distilled water (ddH_2_O) and incubated overnight under dark conditions. The deionized water was replaced with 100 μL reaction solution comprising 20 μM L-012 (TargetMol Chemicals Inc., T22096), 10 μg/mL horseradish peroxidase (Beyotime Biotechnology, P2369), and either flg22 or Agrivax. ROS production was measured using a High Sensitivity Plate Luminescence Detector (BLT Lux-P110). The experiment was performed three times.

### 1.8 Trypan blue staining

The leaves from 4-week-old soil-grown *Arabidopsis* plants were infiltrated with Agrivax solution (at the specified concentration) or DMSO (as a mock control) using a needleless syringe. Following infiltration, the plants were maintained under high-humidity conditions for 24 h. The pretreated leaves were challenged with a suspension of *Pst AvrRpt2* (OD_600_ = 0.002 in 10 mM MgCl_2_) using a needleless syringe. The plants were then returned to high-humidity conditions. At the indicated dpi, leaves were collected and fully submerged in 5 mL of freshly prepared trypan blue staining solution (comprising equal parts lactic acid [85% v/v], TE-buffered phenol [pH 7.5–8.0], glycerol [≥99%], and distilled water, containing 10 mg/mL trypan blue). The samples were incubated at room temperature for at least 30 min (up to 1 h) to allow the dye to penetrate dead tissue. To visualize the results, the staining solution was replaced with absolute ethanol for overnight destaining. The ethanol was refreshed multiple times until the chlorophyll was completely cleared, leaving only the blue-stained necrotic areas visible.

### 1.9 Electrolyte leakage assay

To quantify cell death and membrane integrity, an electrolyte leakage assay was performed using 4-week-old soil-grown *Arabidopsis* plants. The leaves were infiltrated with a specified concentration of Agrivax or DMSO (mock control) using a needleless syringe. After 24 h of incubation under high-humidity conditions, the pretreated leaves were challenged with *Pst AvrRpt2* (OD_600_ = 0.002 in 10 mM MgCl_2_) and returned to high-humidity conditions. At the indicated dpi, the leaves were rinsed twice with deionized water to remove surface contaminants. Leaf discs (0.5 cm in diameter) were excised and floated in 10 mL of deionized water with gentle agitation at room temperature for 4 h. The initial conductivity (C1) of the solution was measured using a conductivity meter. To determine total conductivity (C2), the samples (including leaf discs) were autoclaved and allowed to cool to room temperature before a second measurement was taken. Relative electrolyte leakage was calculated using the following formula: Electrolyte Leakage (%) = (C1/C2) ∗ 100.

## CRediT authorship contribution statement

**Changlong Liu:** Conceptualization. **Qingqiu Shi:** Conceptualization. **Ning Bai:** Formal analysis. **Xuechun Hu:** Data curation. **Yajin Ye:** Conceptualization. **Lijuan Zhou:** Conceptualization.

## Declaration of competing interest

The author declares no competing interests.

## Data Availability

All data supporting the findings of this study are available in this paper and supplementary information.
