# Supplementary material for: Agrivax: A chemical inducer of plant immunity identified by high-throughput screening
Source: aBIOTECH. 2026 Mar 19;7(2):100038. doi: 10.1016/j.abiote.2026.100038 (PMC13090639; doi:10.1016/j.abiote.2026.100038)
Supplement: Multimedia component 1 [file mmc1.pdf]

# Supplementary Figure 1

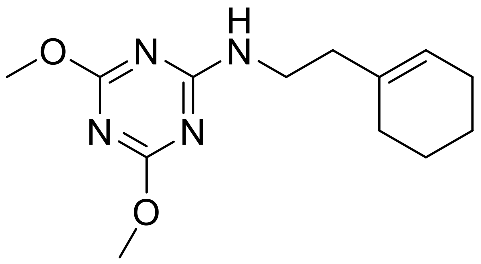

**Agrivax**

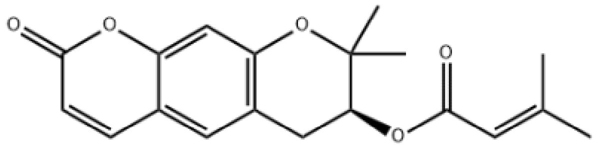

**Decursin**

Tanimoto Coefficient: 0.0000

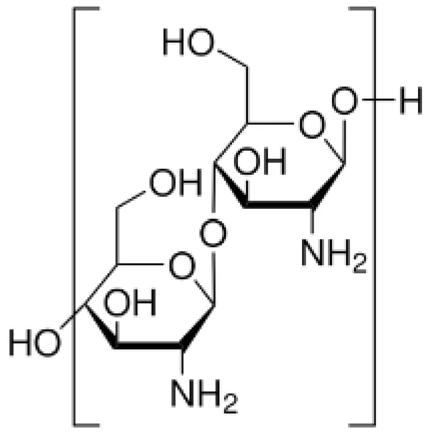

**Chitosan**

Tanimoto Coefficient: 0.0000

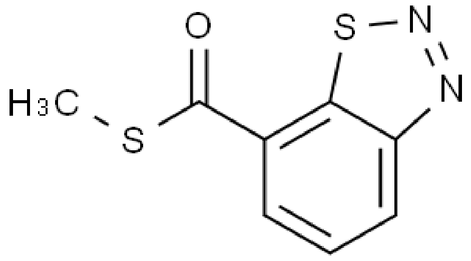

**Benzothiadiazole**

Tanimoto Coefficient: 0.0000

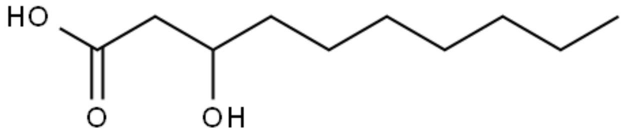

**3-OH-C10:0**

Tanimoto Coefficient: 0.0000

**Supplementary Figure 1.**

Chemical structures and Tanimoto coefficient calculations comparing Agrivax to known immune elicitors, including Decursin, Chitosan (COS), Benzothiadiazole (BTH), and 3-OH-C10:0. Tanimoto coefficients of 0.0000 confirm that Agrivax is a structurally unique molecule compared to these existing elicitors.

# Supplementary Figure 2

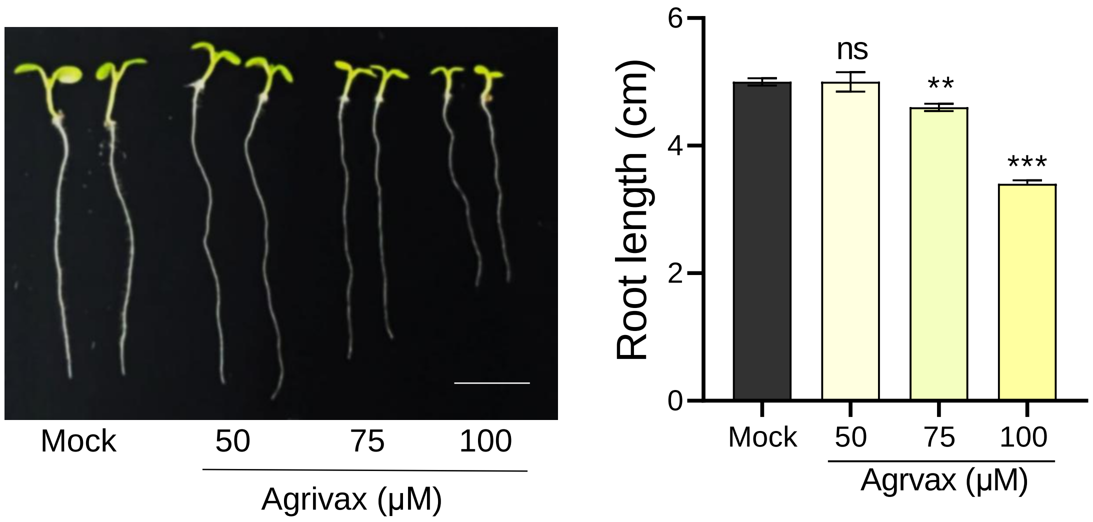


**Supplementary Figure 2.** Dose-dependent effect of Agrivax on Arabidopsis root growth. (A) Representative images of 7-day-old seedlings grown on vertical plates containing different concentrations of Agrivax (50, 75, 100 μM). (B) Quantification of primary root length. While 50 μM Agrivax shows no significant (ns) inhibition, higher concentrations significantly reduce root growth, reflecting a growth-defense tradeoff. Error bars represent SEM (n = 5); **P < 0.01; ***P < 0.001 (2-tailed Student's t-test). Scale bar = 1.5 cm.

# Supplementary Figure 3

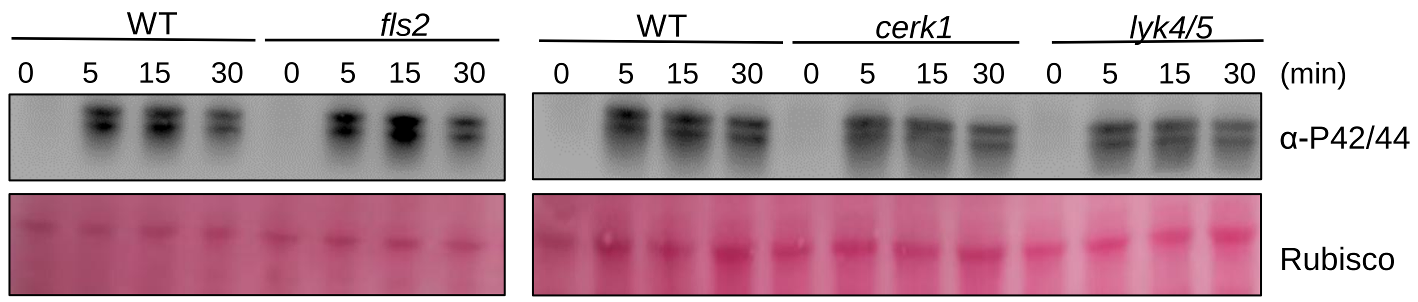


**Supplementary Figure 3**

Western blot analysis of MAPK activation in Wild-Type (Col-0) and PRR-deficient mutants (*fls2*, *cerk1*, and *lyk4/5*) following Agrivax treatment. 7-day-old seedlings were collected at 0, 5, 15, and 30 minutes post-treatment. α-P42/44 antibody detects phosphorylated MAPKs; Rubisco is shown as a loading control.

## Supplementary Table 1

The predicted solubility of Agrivax using SwissADME

| Solute | Solvent | Temperature | Predicted Solubility |
|---|---|---|---|
| Agrivax | H2O | 25 ℃ | 0.13 M |
| Agrivax | Ethonal | 25 ℃ | 0.23 M |
| Agrivax | DMSO | 25 ℃ | 0.34 M |
